# A New Technique for Direct Fabrication of Fiber-Reinforced Composite Bridge: A Long-Term Clinical Observation †

**DOI:** 10.3390/dj8020048

**Published:** 2020-05-10

**Authors:** Matías Ferrán Escobedo Martínez, Samuel Rodríguez López, Jairo Valdés Fontela, Sonsoles Olay García, Mario Mauvezín Quevedo

**Affiliations:** 1 Department of Odontología Integrada de Adultos, Faculty of Odontology, University of Oviedo, 33006 Oviedo, Spain; samuelopezgij@gmail.com (S.R.L.); olaymaria@hotmail.com (S.O.G.); mauvezinmario@uniovi.es (M.M.Q.); 2 Private Practitioner, 33003 Oviedo, Principado de Asturias, Spain; valdesfontela@me.com

**Keywords:** fiberglass bridge, fiber-reinforced composite bridge, minimal invasive preparation

## Abstract

The use of fiberglass in dentistry has increased due to the improvements in the development of adhesive techniques reducing the cost of treatment and avoiding abutment tooth craving. The present study aims to evaluate the clinical usefulness of the new technique to fabricate a direct fiber-reinforced composite bridge (FRCB) over a long period of time. Twenty-one FRCB were performed with the new direct technique on 21 patients with a mean age of 58.85 years and female predominance in the Faculty of Dentistry of Oviedo (Spain). The framework design releases the embrasures allowing adequate interproximal brushing, avoidance of periodontal disease and interproximal caries. A baseline examination was performed and the patients were examined regularly at six-month intervals (nine years’ follow-up). The restorations were also evaluated by an examiner using parameters to check their stability, longevity and the lack of periodontal disease. The most frequent location was the maxillary premolar region and the purpose of the restorations was to give a definitive bridge in 100% of the patients. Only one total debonding of the prostheses was detected during the observation period at 24 months and three partial adhesive–cohesive veneering composite fractures at the pontic after 60, 72 and 84 months, respectively. Kaplan–Meier was performed to detect the overall survival rate of the restorations at the end of the follow-up. Nine-year survival rates for the FRCB was 95.2%. All the cases had a clinically acceptable periodontal condition and an interproximal absence of caries in the abutment teeth. Currently, this type of restoration allows a minimally invasive aesthetic and is an affordable procedure, being a good alternative to other types of treatments.

## 1. Introduction

Currently, prosthetic replacements of anterior or posterior edentulous segments present an aesthetic challenge to clinicians. A variety of therapeutic modalities, from implants to conventional fixed partial dentures or even removable partial dentures, have been used for the replacement of missing teeth. Each of these techniques has advantages and disadvantages related to their durability: the overall complexity of the cases, their cost (both biological and financial) and challenges to oral hygiene after the treatment [1,2].

Fiber-reinforced technology was initially used as a splint material for periodontally involved teeth and to stabilize avulsed teeth [3]. Nowadays, it is used to replace anterior or posterior teeth [4,5,6,7]. Selection criteria for the use of fiber-reinforced composite bridge (FRCB) depend on the location in the mouth and occlusal forces. Several studies have suggested evaluating the health of abutment teeth, the lack of periodontal disease or well treated periodontal disease which is under control [8,9] and the lack of parafunctional habits [10,11]. Additional criteria that must be evaluated before selecting this treatment option include uncontrolled medical history which negatively influences oral health [9], such as diabetes [8], the length of the edentulous space, loading on the pontic tooth [12], the type and form of presentation of the fiber as well as the technique that we use.

Regarding the type of fiber-reinforced, glass fiber or the ultra-high molecular weight polyethylene (UHMWPE) fiber is normally used. These materials have shown better performance as the core of FRCBs [4,5]. In relation to its presentation, the most frequent are the meshes that can be impregnated in bonding to make their placement easier [13].

Fiber-reinforced types are heterogeneous and anisotropic, meaning their properties strongly depend on the direction in which they are tested relative to their fiber orientation. For unidirectional fiber-reinforced types in which the fibers run parallel and in one direction, properties are highest in the direction parallel to the fibers and lowest in the direction perpendicular to the fibers. To give the fiber better properties, they create meshes with multiple fiber orientations several times. This can be achieved in one of two ways: either by placing unidirectional fibers in multiple directions or by using a braided or woven fabric [14].

At this moment, FRCB is performed with two different types of techniques (direct or indirect techniques). The direct technique allows the prosthesis to be done in a single appointment, excluding laboratory needs and reducing the cost of treatment. Even so, it is necessary that the technician has great knowledge of fiber management and preparation of the operative area (isolation with the rubber dam) [2,15]. On the other hand, the indirect FRCB technique is performed in the laboratory. The success of this technique is based on the skills the laboratory technician has with the fiber and adequate isolation of the operative area and cementation of the FRCB by the technician. The disadvantages of this method are the cost and the number of appointments [7,16].

If we take all these considerations into account, this material offers an attractive alternative for the treatment for the replacement of tooth loss. On the one hand, this type of prosthesis allows easy repairs in a single appointment such as composite chipping, without the help of a laboratory technician. Other advantages include reduced cost (biological and financial) compared with conventional bridges, absence of metal allergies and a natural feeling [2,17].

The aim of this clinical retrospective study was to analyze the survival rate and rule out the possible complications that this new direct technique for performing FRCB can present. We believe that this proposed aim will allow us to discard the null hypothesis that the nine-year survival rate of follow-up of the FRCB performed with this new technique has been deemed unacceptable.

## 2. Material and Methods

Between June to July 2010, 21 patients with a complaint of an anterior or posterior single missing tooth were randomly treated with an FRCB in the Faculty of Dentistry of Oviedo, University of Oviedo, Spain. The main reason for the treatment with FRCB was the need for a low-priced fixed prosthesis and the need to save tooth substance. We obtain signed informed consent from patient of the pictures in this paper.

The selection criteria of the patients included:

1. Maintenance of good oral hygiene;
2. No parafunctional habits;
3. The absence of large restorations or caries lesions on the abutment teeth;
4. No periodontal disease of the abutment teeth (periodontal evaluation procedures: the sulcus bleeding index [18], Silness–löe plaque index [19] and the probing depths).

The sample size depended on the number of patients who requested a first-time visit at the Faculty of Dentistry of Oviedo during the patient acquisition phase for fifth-year students for the following year.

This study did not require previous animal experimentation as the materials which were used for the realization of the FRCB are very common in dental treatments of the 21st century. At the same time, approval from an ethical committee was not necessary for the realization of this case series.

**Figure 1:**
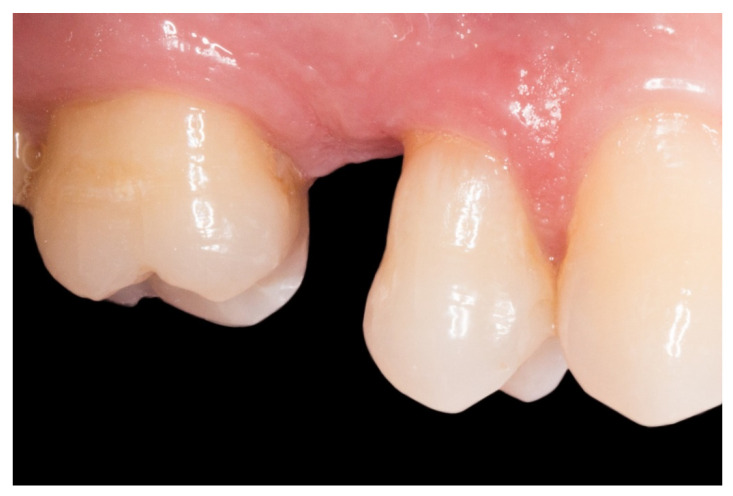
A posterior maxilla edentulous space.

**Figure 2:**
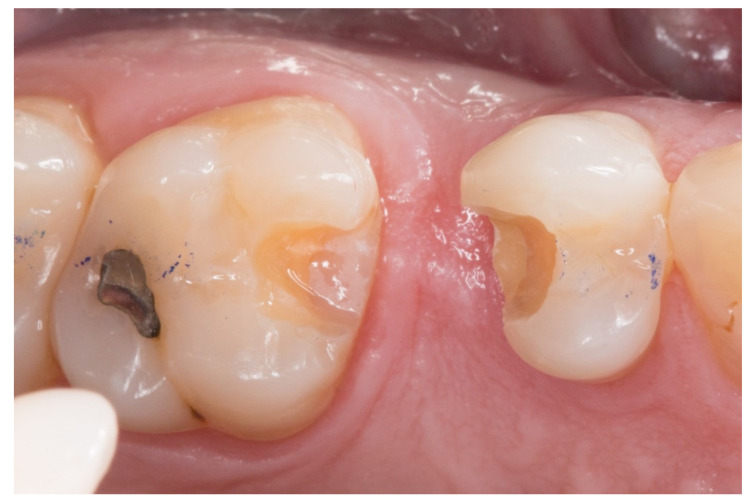
The proximal faces of the adjacent teeth delimiting the edentulous space carved up to the level of the ideal point of contact (inlay cavities).

**Figure 3:**
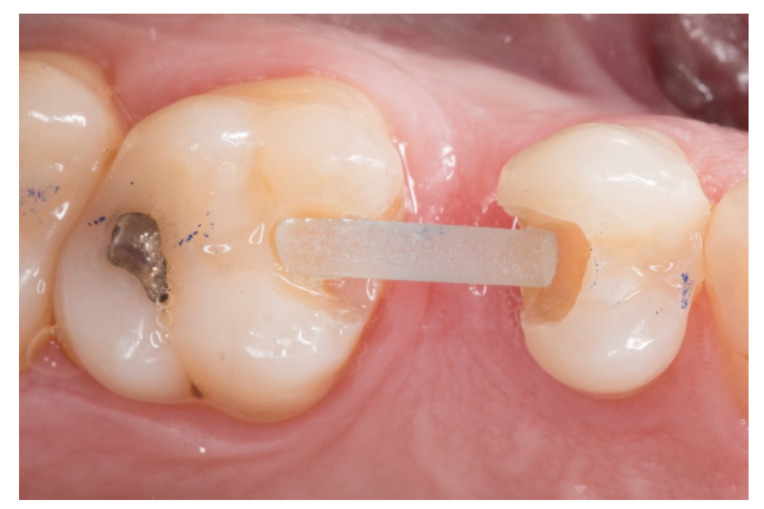
The transverse structure of the future fiber-reinforced composite bridge (FRCB).

**Figure 4:**
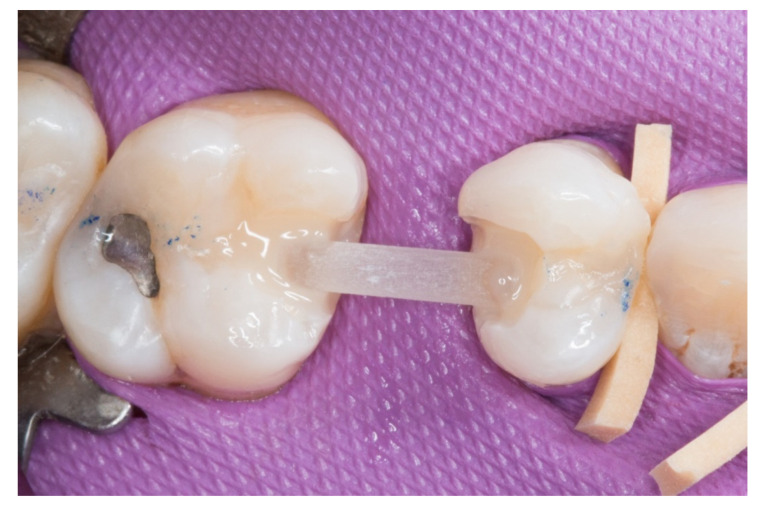
The horizontal fiberglass pin bonding to the adjacent teeth.

**Figure 5:**
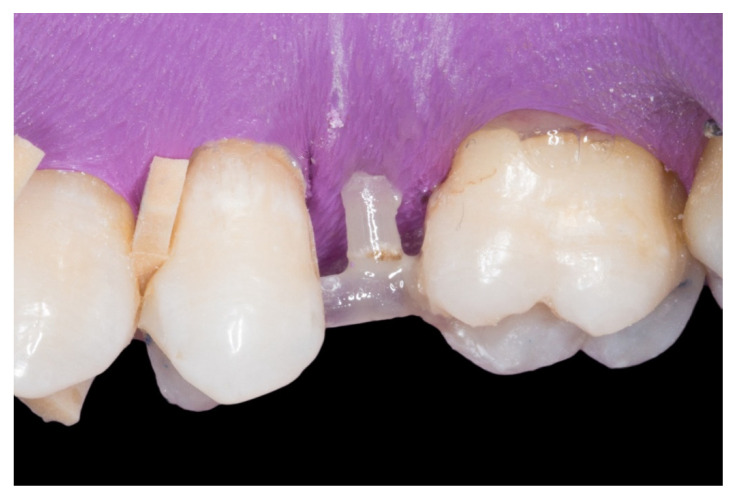
“T” shape of the fiber-reinforced composite bridge.

**Figure 6:**
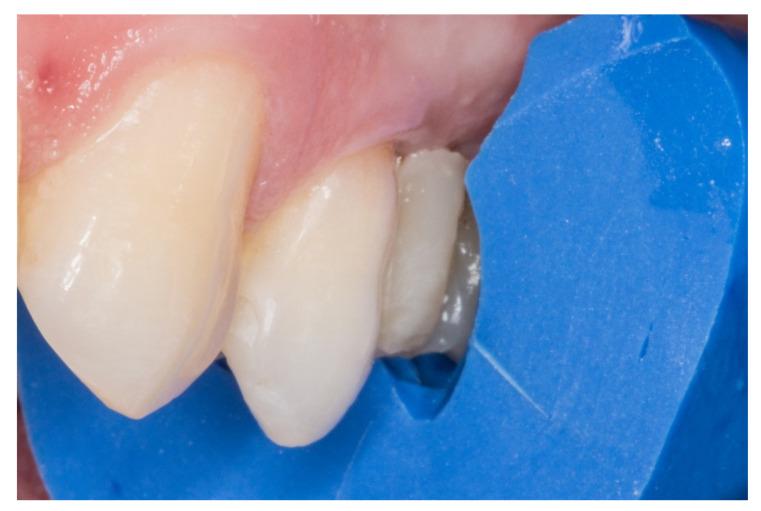
The crown of the pontic and the occlusal surfaces of the inlay preparations were formed incrementally using a key silicone.

**Figure 7:**
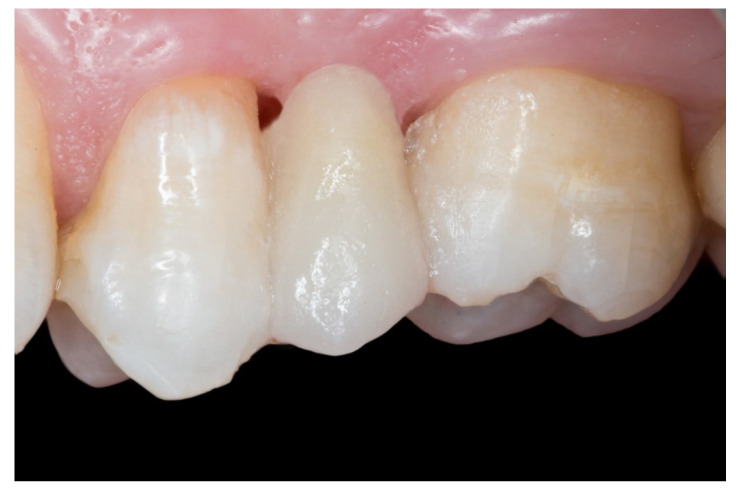
The FRCB after the occlusal adjustment and composite polishing.

The follow-up of the restorations reported here stopped by the end of June 2019. Restoration procedures were explained to the patients, and the 21 patients who accepted this protocol were treated by 2 operators as follows (Figure 1, Figure 2, Figure 3, Figure 4, Figure 5, Figure 6, Figure 7 and Figure 8):

1. The new technique in “T” started once the silicone impressions of the patient were taken for both arches, and an interocclusal registration in the maximal intercuspation position was obtained, in order to prepare (on an articulator) the wax-up and the silicone keys for the tooth replacement [20]. Figure 1 represents the initial state of a case collected in the study.

2. The proximal faces of the adjacent teeth delimiting the edentulous space were carved up to the level of the ideal point of contact (inlay cavities), to position the future horizontal structure of the fiberglass bridge (Figure 2).

3. The next step consisted of the horizontal fiberglass pin (Rebilda^®^ Post GT, VOCO GmbH, Cuxhaven, Germany) bonding to the adjacent teeth. Firstly, when the isolation of the operative field with the rubber dam was made, the fiberglass pin was adjusted to size and silanization according to the instructions by the manufacturer. Later, the inlay cavities were etched with Ultra-etch^®^ (Ultradent Products Inc, South Jordan, UT, USA) for 20 s, rinsed for 10 s and dried for 10 s. The etched surfaces were covered with a layer of a universal adhesive resin (Prime & Bond^®^ NT, Dentsply Sirona Inc., York, PA, USA), thinned using a brush, and cured for 20 s with a light- polymerizing unit. A flowable resin (Tetric Evo flow^®^, Ivoclar Vivadent AG, Schaanwald, Liechtenstein) was used to cover the inlay cavities (inlays retainers) and the fiberglass pin to shape the transverse structure of the future bridge (Figure 3 and Figure 4).

4. Afterwards, the pontic core was reinforced with a vertical pin fiberglass structure, which was followed by the same adhesion process mentioned above, obtaining a “T” shape (Figure 5).

5. The crown of the pontic and the occlusal surfaces of the inlay preparation were formed incrementally using key silicone (pontic’s shape) and nanohybrid composite resin (Tetric EvoCeram^®^, Ivoclar Vivadent AG Schaan, Liechtenstein, Figure 6).

6. Finally, when the FRCB were complete, the occlusion was checked, premature contacts were relieved and the restorations were polished with a composite finishing and polishing kit (Figure 7).

**Figure 8:**
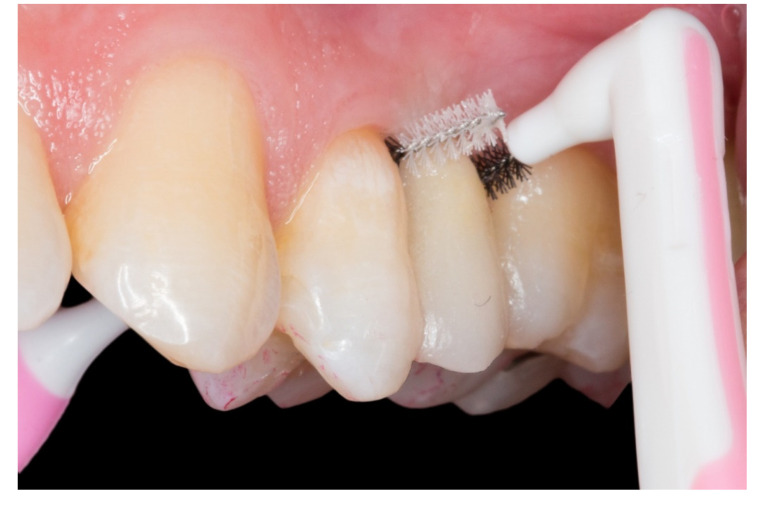
The embrasures are released allowing adequate interproximal brushing, avoiding periodontal disease and interproximal caries.

On this “T” technique the embrasures are released allowing an adequate interproximal brushing, avoiding periodontal disease and interproximal caries (Figure 8).

Baseline examination was performed 24 h after the restoration placement. The patients were examined regularly every 6 months and the vitality of the abutment teeth was checked and noted along with the subjective complaints of the patients. Periodontal evaluation was performed at each recall and the importance of oral hygiene was emphasized when needed. The restorations were also evaluated by using parameters to check their stability and longevity: retention, marginal and pontic discoloration, secondary caries, morphological consistency and marginal adaptation for the abutment teeth and fracture of the FRCB.

Partial or total debonding of the FRCB and framework fracture were considered treatment failures. The survival estimation method of Kaplan–Meier was performed to detect the overall survival rates of the restorations made of each fiber reinforcement at the end of the follow-up.

## 3. Results

Twenty-one FRCB were performed on 21 patients with a mean age of 58.85 years and female predominance (14 patients). The most frequent area was the maxillary premolar region followed by the maxillary incisor sector and the purpose of the restorations were as a definitive bridge in 100% of the patients (Table 1).

Two patients did not complete the observation period (exitus at 54 and 72 months).

Only one total debonding of the prostheses was detected during the observation period at 24 months. This prosthesis was later replaced with a conventional full-coverage fixed prosthesis.

Three partial adhesive–cohesive veneering composite fracture at the pontic. They occurred either in the occluso-buccal or occluso-lingual area of the pontic after 60, 72 and 84 months, respectively, and one FRCB needed polished at 72 months. They were repaired and polished in situ (Table 2).

All the cases had a clinically acceptable periodontal condition due to the embrasures and were released allowing adequate interproximal brushing. The patients reported no caries, no temperature or pressure sensitivity during the follow-up and all the abutment teeth were vital at the final examination appointments.

**Figure 9:**
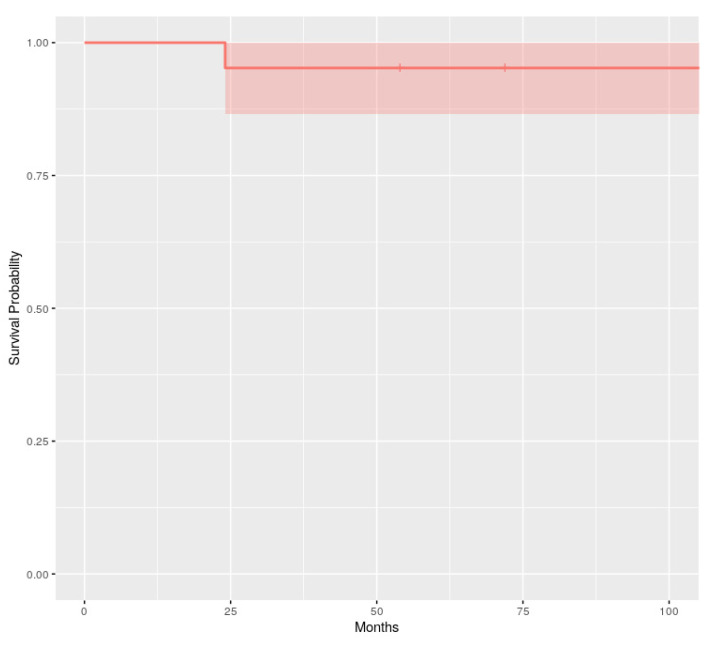
Kaplan–Meier survival curve for the FRCB (overall survival estimation and confidence interval).

As shown in the Kaplan–Meier curves, the overall survival probability at 108 months for the FRCB was 0.952 (95% confidence interval: 0.866–1, Figure 9).

## 4. Discussion

Loss of teeth can psychologically and socially damage the patient, and this trauma can be minimized by immediate replacement of the teeth, preferably using a fixed prosthesis. FRCB offers a good option for restoring a missing tooth which is less invasive than conventional full-coverage fixed prosthesis. However, the difficulty some practitioners have in achieving good pontic esthetics in the directly fabricated types of FRCB often proves a barrier to their implementation [2].

If we perform an analysis of published literature on these types of restorations, we can see multiple types of fiberglass presentations (meshes, pins and others), as well as an endless combination of these with restorative materials to make the pontic, even natural teeth. On the other hand, FRCBS are used with a very broad age spectrum. This is because at very early ages they are used as temporary prostheses after orthodontic treatment or after a non-restorable dental trauma [21,22] until the end of bone growth and in adults, as a provisional prosthesis after implant placement with a definitive end [23], as in the case of our work.

Nowadays, the incisive localization is indicated as the most frequent region for this type of restoration [2,10,23]. Although, we can find them in premolar and even molar regions, as long as the indications and adhesive protocol are respected [2]. Our clinical study is an example of this trend due to the premolar region being the most frequent location.

The length of the edentulous space is another consideration to keep in mind. Large edentulous spaces (more than one tooth) would be a contraindication for this type of prosthesis, since it would increase the probability of fracture or failure of the FRCB. For this reason, it is important to consider a patient with small edentulous spaces and very soft contact points on the FRCB pontic.

Failure by partial or total debonding of the FRCB and framework fracture, as witnessed under clinical condition, is a well-known phenomenon in dental literature [24]. Various authors [11,22,25] described this mode of failure as a two-phase failure pattern, consisting primarily of cracking and chipping of the veneer layer, followed by adhesive failure between the veneer and glass fiber material. Spinas et al. [26] reported similar failure results after five years of use in 3 of 41 FRCB. In contrast, Cenci et al. [27] reported a 34.2% success rate for the 13 FRC-Bridges included in a clinical study with a mean follow-up of 96 months.

In relation to the fiberglass pin, this should have a higher fracture resistance than the average masticatory forces and should contain physical properties such as an elastic modulus, compressive strength, and coefficient thermal expansion similar to the tissue (dentin) that we seek to replace as the core of the FRCB [28,29]. For this reason, we believe that this type of fiberglass pin has the appropriate characteristics. If we add the right type of fiberglass pin diameter according to the region where the FRCB is located (anterior sector: Φ 0.8 to 1 mm: posterior sector: Φ 1 to 1.4 mm), we will achieve greater success in our treatment. In 2017, Saritha et al. [30] carried out a study comparing the resistance to a fiberglass pin fracture (similar to our what we used) versus a zirconia pin or a carbon pin. The authors confirmed that the zirconia pin was the most resistant, but that it had more disadvantages related to the handling of the material and the modulus of elasticity of the tissue to be replaced (dentin) compared to the pins made of fiberglass. For this reason, they concluded that considering the advantages and disadvantages, fiberglass pins were the optimal choice compared to zirconia or carbon pins. This allows us to confirm that the use of fiberglass pins as the FRCB’s core would be a good choice. We opted for this material and reinforced it with a second vertical section of a fiberglass pin in order to give the core greater resistance.

Recently, Goguta et al. [12] published an article with six years of follow-up on the resistance of FRCB. In it, they compared two types of FRCB in relation to the type of retention of the prosthesis (wings or inlay retainers). As a result of the study, it was concluded that FRCB with inlay retainers had a lower failure rate, their survival rate being very similar to our study (94.47% success rate). With this work, we reinforce our idea that this type of retention (inlay retainers) allows greater survival of the FRCB, due to a higher volume of the retention structure. On the other hand, we have to recognize that in order to achieve this retentive volume, greater carving of the abutment teeth is necessary.

Regarding the periodontal aspect and the development of interproximal caries, few articles have been written on this very important aspect [2]. We consider it to be one of the main problems in this type of restoration, due to the need to build a strong and solid framework. Practitioners invade periodontal tissue, generating plaque accumulation causing chronic periodontal inflammation and the development of interproximal caries in the abutment teeth. This technique that we propose uses inlay retainers and two fiberglass pins which form the “T” structure is very solid and stable, allowing the release of embrasures and enabling adequate interproximal brushing. For this reason, as mentioned in the article by Izgi et al. [2], it is important to have follow-ups every six months in order to control the absence of complications in the FRCB and to check up on the abutment teeth. In our opinion, adequate brushing techniques, flossing properly and interproximal brushing should be taught by the practitioners to avoid complications in abutment teeth (interproximal caries and periodontal inflammation).

Another problem that practitioners found in this type of rehabilitation was its durability. Normally they were designed to be used provisionally, as the adhesion techniques were underdeveloped due to the fragility of the fiberglass. At present, they are used for definitive restorations, because these problems practically do not exist and the survival has extended exceeding up to even six years [12]. In our study with nine years of follow-up, the overall survival was 0.952 at the end of the study.

On the other hand, we recognize the limitations of our study in relation to the sample size and the lack of a control group. Therefore, the results can only be considered preliminary. It would be interesting to increase the number of patients in the future, as well as the number of dental schools to collaborate with in order to see if our results can be extrapolated to a large sample with a control group.

In conclusion, on the one hand, the results obtained discard the null hypothesis as the nine-year survival of the FRCB is acceptable (95.2% survival rate).

On the other hand, this technique could solve two main drawbacks with this type of restoration:

1. The periodontal inflammation aspect on the pontic area: the framework design releases the embrasures allowing adequate interproximal brushing, avoiding periodontal disease and interproximal caries.
2. Long term survival: this proposed protocol which uses inlay retainers and a core with two “T” shaped pins reinforces and stabilizes the FRCB, allowing greater survival over time.

## Figures and Tables

**Table 1:** Description of the patients and the prostheses.

*n* = 21	Age (Years)	Gender (Male/Female)	Location of Prosthesis (Abutment Teeth *n*_1–_*n*_2_)		Type of Framework
1	83	M	Maxillary left	(3–5)	Glass fiber
2	36	M	Maxillary right	(3–5)	Glass fiber
3	57	M	Maxillary left	(4–6)	Glass fiber
4	35	M	Maxillary right	(1–3)	Glass fiber
5	77	M	Mandibular left	(4–6)	Glass fiber
6	50	M	Maxillary left	(3–5)	Glass fiber
7	67	F	Mandibular right	(1–3)	Glass fiber
8	67	F	Maxillary right	(3–5)	Glass fiber
9	45	M	Mandibular left	(5–7)	Glass fiber
10	25	F	Maxillary right	(4–6)	Glass fiber
11	36	M	Mandibular right	(5–7)	Glass fiber
12	62	M	Mandibular right	(1–2)	Glass fiber
13	88	M	Maxillary right	(3–5)	Glass fiber
14	64	M	Maxillary right	(1–3)	Glass fiber
15	35	F	Maxillary right	(4–6)	Glass fiber
16	53	M	Maxillary right	(1–3)	Glass fiber
17	85	M	Maxillary right	(2–4)	Glass fiber
18	87	F	Maxillary left	(3–5)	Glass fiber
19	38	F	Maxillary right	(3–5)	Glass fiber
20	65	M	Maxillary right	(1–3)	Glass fiber
21	81	F	Maxillary right	(3–5)	Glass fiber

**Table 2:** Survival results of the prosthesis at the end of the follow-up.

*n* = 21	Partial or Total Debonding of Prosthesis	Fracture Area of Prosthesis (Months)			Survival Time (Months)
		Mesial Abutment	Pontic	Distal Abutment	
1	-	-	-	84 M	108 M
2	-	-	-	-	108 M
3	-	-	-	-	108 M
4	-	-	-	-	108 M
5	-	-	-	-	108 M
6	-	-	-	-	108 M
7	-	-	-	-	108 M
8	-	-	-	60 M	108 M
9	-	-	-	72 M	108 M
10	-	-	-	-	108 M
11	-	-	-	-	108 M
12	-	-	-	-	108 M
13	-	-	-	-	72 M (EXITUS)
14	-	-	-	-	108 M
15	-	-	-	-	108 M
16	Total debonding	-	-	-	24 M
17	-	-	-	-	108 M
18	-	-	-	-	54 M (EXITUS)
19	-	-	-	-	108 M
20	-	-	-	-	108 M
21	-	-	-	-	108 M

M = months; - Indicates the absence of partial or total debonding of the prosthesis or fracture area of the prosthesis.
